# Incorporating temporal information during feature engineering bolsters emulation of spatio-temporal emergence

**DOI:** 10.1093/bioinformatics/btae131

**Published:** 2024-03-05

**Authors:** Jason Y Cain, Jacob I Evarts, Jessica S Yu, Neda Bagheri

**Affiliations:** Department of Chemical Engineering, University of Washington, Seattle, WA 98195, United States; Department of Biology, University of Washington, Seattle, WA 98195, United States; Department of Biology, University of Washington, Seattle, WA 98195, United States; Department of Chemical Engineering, University of Washington, Seattle, WA 98195, United States; Department of Biology, University of Washington, Seattle, WA 98195, United States

## Abstract

**Motivation:**

Emergent biological dynamics derive from the evolution of lower-level spatial and temporal processes. A long-standing challenge for scientists and engineers is identifying simple low-level rules that give rise to complex higher-level dynamics. High-resolution biological data acquisition enables this identification and has evolved at a rapid pace for both experimental and computational approaches. Simultaneously harnessing the resolution and managing the expense of emerging technologies—e.g. live cell imaging, scRNAseq, agent-based models—requires a deeper understanding of how spatial and temporal axes impact biological systems. Effective emulation is a promising solution to manage the expense of increasingly complex high-resolution computational models. In this research, we focus on the emulation of a tumor microenvironment agent-based model to examine the relationship between spatial and temporal environment features, and emergent tumor properties.

**Results:**

Despite significant feature engineering, we find limited predictive capacity of tumor properties from initial system representations. However, incorporating temporal information derived from intermediate simulation states dramatically improves the predictive performance of machine learning models. We train a deep-learning emulator on intermediate simulation states and observe promising enhancements over emulators trained solely on initial conditions. Our results underscore the importance of incorporating temporal information in the evaluation of spatio-temporal emergent behavior. Nevertheless, the emulators exhibit inconsistent performance, suggesting that the underlying model characterizes unique cell populations dynamics that are not easily replaced.

**Availability and implementation:**

All source codes for the agent-based model, emulation, and analyses are publicly available at the corresponding DOIs: 10.5281/zenodo.10622155, 10.5281/zenodo.10611675, 10.5281/zenodo.10621244, respectively.

## 1 Introduction

Grand challenges in biology have required tools with increasingly higher resolution and throughput while also sampling across spatial and temporal axes (Bindea *et al.* 2013, Lähnemann *et al.* 2020, Johnson *et al.* 2021, Eftimie 2022). In particular, dynamic data from live cell imaging is positioned to become the next ‘omics’ (Lelek *et al.* 2021, Bagheri *et al.* 2022) with lower-level resolution data, like scRNAseq, contributing a more nuanced understanding of individual system components across space (Hwang *et al.* 2018, Heumos *et al.* 2023). Our ability to utilize high resolution data has often lagged behind our ability to generate said data (Ji *et al.* 2017, Lähnemann *et al.* 2020, Eftimie 2022). Parity between computational and experimental approaches will allow researchers to synergistically utilize computational models to explain nonintuitive observations, identify ‘rules of life,’ and design model-driven experiments that test new hypotheses. This parity will identify both temporal and spatial components that are fundamental to specific biological systems *a priori* (Möller and Pörtner 2021, Eftimie *et al.* 2023). In order to address this knowledge gap, we interrogate the use of statistical emulation to estimate emergent behavior in a comprehensive analysis of a high-resolution agent-based model.

Computational models capable of characterizing cellular dynamics over time and space across multiple scales are fundamental to scientific progress. Agent-based models (ABMs) have gained popularity due to their ability to simulate populations of heterogenous agents dynamically over time and in space in order to predict system-level emergent properties (Shi *et al.* 2014, Vodovotz and An 2019, West *et al.* 2023). ABMs are designed using the behavior and interactions of individual agents (usually cells) in an evolving spatial and temporal context (Bonabeau 2002, Sklar 2007). This rule-based approach is well suited to modeling emergent behaviors, such as the development of multi-cellular systems (Osborne *et al.* 2017, Ghaffarizadeh *et al.* 2018, Corti *et al.* 2021, Pleyer and Fleck 2023) or the spread of infectious diseases (Hunter *et al.* 2018, Gomez *et al.* 2021, Kerr et al. 2021). ABMs can capture the heterogeneity and the stochasticity of biological systems, as well as the bilateral relationship between the local microenvironment and agents’ behaviors (Metzcar *et al.* 2019, Norton *et al.* 2019, Yu and Bagheri 2021). Furthermore, ABMs can be used to investigate the effects of different parameters and conditions on emergent behaviors, providing insights into complex biological processes that may be difficult or impossible to observe experimentally (Yu and Bagheri 2016, Soheilypour and Mofrad 2018, Virgilio *et al.* 2018, Peng and Vermolen 2020, Yu and Bagheri 2021). As such, ABMs are an increasingly important—albeit computationally expensive—tool for understanding complex biological systems and generating testable hypotheses that can inform experimental design.

Statistical emulation (SE), an approach to surrogate modeling, generates statistical models by mapping independent variables (inputs) to dependent variables (outputs) via statistical inference and machine learning (ML) with limited knowledge of the underlying simulation model. Effective SE produces simplified models that are computationally cheaper to analyze, and therefore interrogate, relative to the original model. The flexible pattern recognition of ML provides two key benefits: (i) the emulation model building process codifies selection of requisite inputs to identify dominant statistical patterns; and (ii) the mathematical frameworks involved in pattern recognition—once trained—are easily calculated via sequences of simple mathematical operations. On the contrary, the feature selection and design process required for SE is laborious, and the algorithms demand significant data for adequate performance. Balancing these trade-offs supports effective integration into multi-class models (Cicchese *et al.* 2017, Cess and Finley 2020), sensitivity analyses for complex models (Alden *et al.* 2020), and parameter sweeps of mechanistic models (Vernon *et al.* 2018, Wang *et al.* 2019). Even with these successes, there have been few comprehensive analyses of the application of emulation models to rule-based models designed to simulate tissue dynamics that emerge from temporal and spatial interactions (Alden *et al.* 2020, Kieu *et al.* 2022, Angione *et al.* 2022). SE of ABMs is largely under-explored despite knowing that the computational demands of large multi-scale ABMs can outpace their utility to generate hypotheses between continuous input variables and heterogeneous outputs (Glen *et al.* 2019, Heppenstall *et al.* 2021). Synergistic development of SE frameworks for ABMs would facilitate expedited analyses and provide systematic methods toward hypothesis generation. Understanding the characteristics (e.g. spatial, temporal, etc.) of data required to emulate key ABM dynamics provides a more thorough understanding of the drivers of emergent outcomes.

### 1.1 The tumor microenvironment as a model system

ABMs of the tumor microenvironment have leveraged *in silico* networks to represent the vascular environment (Norton *et al.* 2018, Metzcar *et al.* 2019, Yu and Bagheri 2021). Physical and structural characteristics (e.g. pressure, shear, radius) are often encoded into network elements like edges and nodes (Fredrich *et al.* 2018, 2019). Networks are flexible data structures that can be modified and interrogated, enabling researchers to study interplay among agents and between agents and environments. Functional coupling between the agents and the environment is required to capture experimentally observable divergent emergence like vascular collapse and necrotic core formation (Norton *et al.* 2018, Yu and Bagheri 2021).

Network analysis provides a means to abstract high-resolution, spatial information into summary statistics (Pavlopoulos *et al.* 2011, Koutrouli *et al.* 2020). Network topology and morphology have been used to understand ecological systems (Peterson *et al.* 2013, Modica *et al.* 2021), interrogate biological pathways (Magnano and Gitter 2021), analyze neurological structure (Sporns 2013, Bassett and Sporns 2017, Kok *et al.* 2020), and identify novel treatment (Iadevaia *et al.* 2010). Specifically in tumor development, network analyses are a promising approach to study healthy and pathogenic vascular mimicry and angiogenesis (Amat-Roldan *et al.* 2015, Alves *et al.* 2018, Fouladzadeh *et al.* 2021). Thus, we hypothesize that vascular network analysis enables the encoding of complex network architecture as interpretable inputs for SE.

In this study, we utilize network analysis to construct SE models of ARCADE, an existing agent-based model of a tumor microenvironment with heterogeneous and realistic vascular networks (Yu and Bagheri 2021). Our investigation reveals a limited relationship between spatial network topology characteristics and emergent tumor properties. We demonstrate the efficacy of incorporating temporal evolution of network metrics to predict emergent properties of the system. Leveraging this temporal information, we develop deep-learning models that improve the predictive power of emulators. Our results highlight the role of temporal dynamics in understanding and predicting emergent properties of diverse cell populations that evolve from lower-level spatial and temporal processes.

## 2 Materials and methods

### 2.1 Data and code availability

All source code for the ARCADE ABM v2.4 is publicly available on Zenodo at 10.5281/zenodo.10622155. The codebase used for emulation is publicly available on Zenodo at 10.5281/zenodo.10611675, and the scripts used to perform analyses and generate figures reported in this paper are also publicly available on Zenodo at 10.5281/zenodo.10621244.

## 3 ARCADE

All ABM simulations and model analyses were performed similar to those described in a previous publication (Yu and Bagheri 2021) using ARCADE v2.4. ARCADE is an on-lattice, agent-based model designed to represent tissue and tumor development. Agents in ARCADE represent tissue cells that can take on seven cell states—quiescent, migratory, proliferative, apoptotic, necrotic, senescent, and undecided. A cell’s transition between states is determined by accounting for their current properties (e.g. age, size), relationship to other agents (e.g. crowding), and local environmental conditions (glucose, oxygen, and TGFα). Each agent contains a metabolism module tracking the conversion of nutrients into energy and cell mass, and a signaling module comprising a ligand-sensing network guiding cell decisions between proliferative and migratory states. For instance, the intracellular signaling state impacts the individual cell’s probability between proliferation and migration. The resulting cellular states determine the discrete behavior performed by the cells at each simulation tick (one minute) within a hexagonal grid. A more detailed description of agent states, rules, and modules can be found in Yu and Bagheri (2020).

The simulation environment is described by a higher resolution triangular lattice containing the concentration of molecules (oxygen, glucose, and TGFα). The molecules diffuse through the environment according a diffusion partial differential equation model given by:

$$\begin{array}{c} \frac{\partial C}{\partial t}=D\nabla^{2}C \end{array}$$

The dynamic vasculature model is represented as a graph data structure, utilizing graph embeddings to represent hemodynamic properties (shear stress, circumferential stress, and flow rate) of the capillaries. These properties are derived from modeling capillary flow through each individual edge with fixed conditions at the originating artery and vein roots. Vascular structure and function change over time based on their coupling with cell agent populations. Functional stresses from tissue cells lead to vascular remodeling, changing vessel radius and wall thickness as a function of hemodynamic properties and metabolic demand. Cancer cells degrade the vasculature by removing components; this removal can lead to larger scale disruption of vascular structure, as functional vasculature requires perfusion. A more detailed description of the environment, dynamic vasculature initialization, and subsequent physical modeling can be found in Yu and Bagheri (2021).

Two agent populations, healthy and cancer cells, exist in the simulations. *In silico* cancer cell populations exhibit hallmarks of cancer: increased crowding tolerance, increased preference for metabolic glycolysis than oxidative phosphorylation, and increased migratory invasiveness versus healthy cell populations (Vander Heiden *et al.* 2009, Hanahan and Weinberg 2011). Toward feasibility and reproducibility, this study does not account for healthy cells evolving to take on cancer hallmarks. The colony and tissue contexts describe simulations comprised of solely cancer cells and a combination of cancer and healthy cell populations, respectively. Prior work highlighted differences in emergent behavior between colony and tissue contexts (Yu and Bagheri 2016, 2021, Prybutok *et al.* 2022b).

### 3.1 ARCADE simulations and workflow

We focus on emulating three emergent tumor properties: activity (instantaneous state of system), growth rate (cumulative temporal behavior), and symmetry (instantaneous spatial state). Activity is the ratio of active to dead cells, describing the degree of necrosis in the tumor. Growth rate is a temporal emergent property that generally describes tumor aggression. Symmetry is a spatial emergent property describing the density and implying the aggressiveness of the tumor. Specific calculations for these properties are detailed in Supplemental information.

The initial vascular structure is the only differing variable between simulations of the same context. Vasculatures are stochastically generated using starting root geometries, detailed in a previous article (Yu and Bagheri 2021); 100 seeds generated a unique vasculature for each starting root geometry and seed combination.

### 3.2 Network analysis and feature extraction

To represent the intricate characteristics of vasculatures within ABM simulations, we employed network analysis. By using a graphical representation of the vasculature, we utilized graph theory metrics as structural and functional features for our ML models. Graph theory provides a number of benefits: it comprises diverse metrics that account for the topological structure of the vasculature; it has mechanisms for specifying vessel importance; and it can quantify overall vessel connectivity. Vascular structure is represented as a network where blood vessels are represented as edges, and where junctions and end points are represented as nodes.

We quantify properties of the *in silico* vasculature and use these features to predict spatial and temporal emergent dynamics (Fig. 4). Aggregate hemodynamics, such as flow and wall thickness, are calculated for each vessel segment and then averaged. Topological features are calculated using the igraph package (Csardi and Nepusz 2006) in Python to create a network representation of the vasculature. Hemodynamic edge features are the same network metrics used to define topological features with one modification: edges in the graph are weighted by hemodynamic properties. Finally, spatial features use distance from the center of the simulation (the tumor seeding location) as edge weights, such that vessels within and closer to the tumor core are weighted more heavily. The supplementary materials (Supplementary Tables S1–S3) offer detailed information about specific graph metrics and how they were obtained.

### 3.3 Statistical emulation

#### 3.3.1 Machine learning models and hyperparameter selection

Python package sklearn was used to build the ML models (Pedregosa *et al.* 2011). All hyperparameters specified are referred to as their respective arguments for each model.

A Sobol random search (Sobol 1976) was used to select the tested hyperparameters during cross-validation using the scipy (Virtanen *et al.* 2020) package in Python. The ranges used for the Sobol random search are detailed in Table 1. Parameter types categorized as ‘linear’ used Sobol indices in the linear space, whereas those types categorized as ‘logarithmic’ used the log of the bounds as the search range. Every discrete parameter value was exhaustively tested in combination with parameter values selected using a Sobol random search on continuous parameter spaces. Each set of Sobol and discrete hyperparameters (Table 2) was used to generate an independent ML model. The model with the best average performance metric (we use the coefficient of determination $R^{2}$) compared across all hyperparameters during cross-validation was used for training and testing. 10-fold cross-validation was used in all cases. The coefficient of determination is defined as $R^{2}=1-\frac{SS}{SS_{\mathrm{tot}}}$, where *SS* is the sum of squares of residuals ML model and $SS_{\mathrm{tot}}$ is the total sum of squares from the mean. $R_{\mathrm{val}}^{2}$ was calculated from ML models evaluated on the validation dataset. The reported $R_{\mathrm{train}}^{2}$ and $R_{\mathrm{test}}^{2}$ were calculated on the training and withheld testing sets after cross-validation. The necessary training times to complete hyperparameter selection are included in Supplementary Table S4.

**Table 1.** Continuous hyperparameters used in cross-validation Sobol search.

		Bounds		
	Hyperparameter	Lower	Upper	Type
MLR	alpha	0.001	1.000	Log
	l1_ratio	0.1	1.0	Linear
SVR	C	0.0001	1.000	Log
	epsilon	0.0	1.0	Linear
RF	n_estimators	1	100	Linear
	max_features	0.01	1.0	Log
	max_depth	1	100	Linear
	min_samples_split	0.01	1.0	Log
	max_samples_leaf	0.01	1.0	Log
MLP	alpha	0.0001	1.000	Log

**Table 2.** Discrete hyperparameters used in cross-validation.

	Hyperparameter	Values
SVR	kernel	linear, poly, rbf, sigmoid
RF	bootstrap	True, False
MLP	activation	identity, logistic, tanh, relu
	hidden_layer_sizes	(5,), (5,5), (5,10), (25,), (25, 25), (25,50), (50,), (50,25), (50,50)

### 3.4 Recurrent neural network architecture and training

In order to account for dynamic network evolution, we trained deep neural networks (NNs) to use time course information to improve predictive performance using the keras (https://keras.io). The purpose of the trained NNs was to predict network evolution from the initial vascular structure in order to increase prediction performance of the emulation models. The neural network utilized a long short-term memory (LSTM) layer, a type of recurrent NN layer capable of capturing sequential patterns (Hochreiter and Schmidhuber 1997). The LSTM layer was followed by 3–4 fully connected layers. Full network topology details are described in Supplementary Fig. S10.

Network topology features from each simulation day were collected and stacked into multivariate time series to facilitate transfer learning of NN parameters. The recurrent NN (RNN) was pre-trained on the full-length time series, encompassing the entire temporal evolution of the network in order to constrain the RNN parameters. To further fine tune the RNN, bootstrapped samples from a subset of the time series (10 days, 5 days, 3 days) were used to sequentially retrain the model (using the previous pretrained deep-learning models as a starting point) and retrain on the initial conditions to provide the ultimate emulator. We applied the best performing model to predict the two week network structures for a reserved test set from only the initial network topology, and then passed the predicted features into the naive ML models (Fig. 4A).

## 4 Results

**Figure 1.**
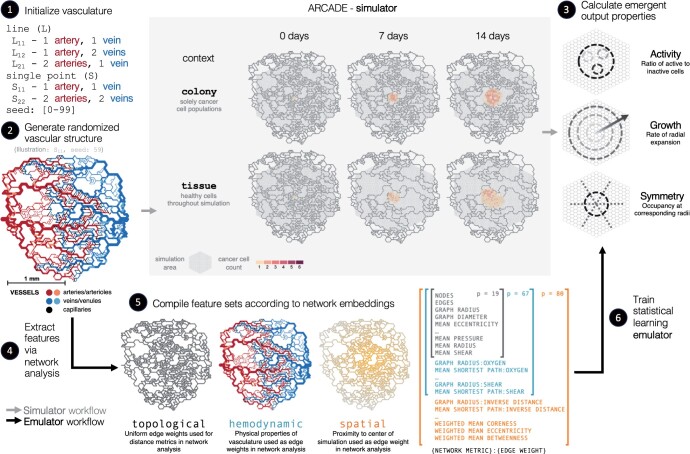
Emulation workflow—a summary of the overall emulation workflow. (1) Vasculature structures are generated based on the a starting root geometry (single point versus line roots, and the number of initializing arteries and veins) and a random seed, where 0–99 were used. (2) ARCADE, an ABM of the tumor microenvironment, receives *in silico* vasculature networks and initial cell population colonies as inputs. ARCADE simulates intra- and inter-cellular interactions among diverse agents to predict the evolution of vascular architecture and function, as well as the evolution of cell populations, over space and time. Two different simulation contexts were used to initialize populations: colony and tissue. (3) Spatio-temporal dynamics are summarized with output metrics that evaluate emergent tumor properties at the end of the simulations: activity, growth, and symmetry. (4) Network metric-based feature sets are extracted from vascular architectures. Nodes represent junctions in the vasculature; edges represent sources of nutrients in the simulation. (5) Feature sets are aggregated based on the information used. Topological features are extracted from the unweighted structure of the network. Hemodynamic features are extracted from attributes of network topologies including hemodynamic characteristics as edge weights. Spatial features account for distance between the information in the network from the center of the simulation. (6) Statistical learning models use network metric-based feature sets to predict emergent tumor output metrics.

The objective was to predict the emergent tumor output metrics by their respective simulation inputs using naive ML algorithms (Fig. 1). Specifically, we used multiple linear regression (MLR), random forest (RF), support vector regression (SVR), and multi-layer perceptron (MLP) to predict emergent tumor output metrics (activity, growth, and symmetry) from the initial condition of the microenvironment vasculature. In general, we use ‘emulators’ to describe those models accepting initial conditions as the sole inputs and we us ‘ML models’ to describe those accepting any other timepoint. The microenvironment was represented by a network analysis of the vascular architecture to provide features for ML models.

Various network analyses generated a suite of feature sets that were used to train the ML models. The topological feature set characterized traditional structural and topological information of the vasculature system (e.g. eccentricity, betweenness, average degrees), as well as mean hemodynamics across the vascular system (e.g. mean pressure, mean shear, mean radius, etc.). The hemodynamic feature set ascribed hemodynamics properties (e.g. flow, pressure, wall thickness) as edge weights to the topological features in order to capture vascular function. The spatial feature set integrated relative locations of edges and nodes from the center of the simulation—which represents the center of the tumor—and accounted for these properties as both additional edge weights and penalties in weighted average calculations. Each feature set is inclusive of the previous set: topological⊆hemodynamic⊆spatial. A comprehensive feature set breakdown is described in Supplementary Tables S1–S3.

### 4.1 Unweighted and hemodynamic-weighted network topologies do not predict emergent tumor output metrics

In order to represent vascular structures in a quantitative format, topological network data from initialization vasculatures were used to train the emulators. Aggregate network metrics (e.g. number of nodes and edges) characterized the size and density of the network. Additional metrics—e.g. average eccentricity, betweenness, and coreness of each node—were used to characterized the average behavior of nodes in the network. This topological feature set does not include spatial node embeddings as a factor in the analysis.

**Figure 2.**
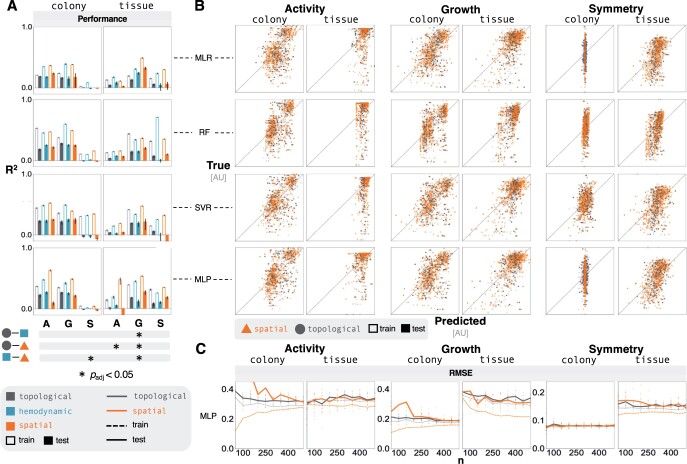
Spatial information does not support emulation—(A) Bar plots show predictive performance of emergent outputs (A: activity, G: growth, S: symmetry) across feature sets for different models (MLR, RF, SVR, and MLP). Feature engineering offered limited improvement. Bar chart values range from −0.1 to 1.0; the horizontal axis is at 0.0. The Bonferroni corrected *P*-values from a two-way ANOVA highlight significant results (noted with asterisks) that have an adjusted *P*-value <.05. (B) Parity plots show differences between the variance in the predicted response and the true response, comparing the topological and spatial feature sets. (C) Additional training data offered diminishing returns on predictive performance of MLP models that were trained on both spatial and topological features. These subplots show the average RMSE as a function of the size of training data. The individual points represent the RMSE from randomized test sets.

Vascular structures represented by initial topological features are not predictive of emergent behavior, resulting in models that exhibit both overfit and underfit characteristics. The coefficient of determination for test data in all topological emulation models is <0.3 (Fig. 2A), suggesting that these models are underfit as a result of the variance in our features not explaining the variance in the emergence. The substantial performance gaps between training and testing results that derive from more complex algorithms (i.e. SVR, MLP) indicate overfitting, despite implementing regularization (Fig. 2A). The best cross-validation performance corresponded with better training performance in simpler algorithms (i.e. MLR, RF), whereas the large majority of cross-validation models had better training metrics than the selected model in the more complex algorithms (Supplementary Fig. S1). We find that the contribution of each feature—based on a permutation importance analysis—was inconsistent between emulators, likely resulting from the collinearity of features and the degree of regularization required for more complex ML algorithms (Supplementary Fig. S2).

Performance is variable as a result of context; activity predictions in colony contexts (Fig. 2A, left) performed more accurately than comparable models in tissue contexts (Fig. 2A, right). On the contrary, emulators that comprise healthy cell background in the tissue context (Fig. 2A, right) reflect more accurate symmetry and growth predictions. Notably, an adequate model—exhibiting a test coefficient of determination over 0.0—for symmetry in the colony context could not be trained.

We then included hemodynamic characteristics as edge weights in the network analysis for network-distance metrics in the distance-based analyses. These hemodynamic features describe higher-resolution physical characteristics of the environment that have clinical implications. For example, pressure-based metrics have been associated with system-level hypertension. (Blanco *et al.* 2017) The resulting weighted network analysis metrics offered limited improvement in prediction accuracy; most results were statistically insignificant relative to the unweighted case, determined by a two-way ANOVA (Fig. 2A). Training and testing performance of hemodynamic emulators experienced diminishing returns as the amount of training data increased (Supplementary Fig. S3).

### 4.2 Network topologies with hemodynamic and spatial information do not predict emergent tumor output metrics

We hypothesized that the spatial variance in vascular structure seeds would account for a significant amount of the variance found in emergent tumor behaviors. This hypothesis was motivated by the finding that vascular collapse, a large-scale dynamic event, substantially impacts the spatial structure of simulations. The consequence of collapse in concert with vessel degradation is required to observe the formation of a necrotic tumor core (Yu and Bagheri 2021). Thus, in order to capture spatial information in the network analysis, we applied a proportional penalty for edges and nodes based on their respective euclidean distance from the center of the simulation (a proxy for the tumor core).

We investigated whether the distance of network nodes from the center of the simulation would explain tumor behavior (Fig. 2A and B). Including spatial features led to negligible, if any, improvement on the emulators’ testing performance. Regularization in the cross-validated emulation models led to predicted targets that spanned less variance in the response variable (Fig. 2), which was indicated by the low coefficients of determination (Fig. 2A). The RMSE of the predicted targets showed minimal improvement as we increased the number of training data (Fig. 2C), suggesting that the poor performance was not a result of insufficient training data; instead, it likely derived from an incomplete representation of the drivers of emergence.

### 4.3 Network analysis of environmental snapshots at later timepoints predict emergent tumor output metrics

In order to interrogate the impact of network evolution on the performance of our ML models, we calculated hemodynamic network metrics for the vascular structure on each simulation day. Features at each timepoint were solely derived from the snapshot at the later vascular architecture. These temporally dynamic features were then used to predict emergent tumor dynamics.

**Figure 3.**
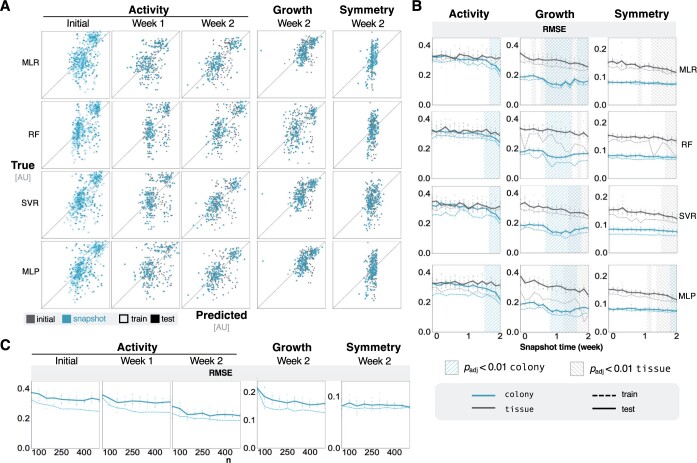
Temporal information improves accuracy of ML models—(A) Parity plots show the predictive performance of colony context ML models that were trained on exclusively on features from later timepoints. The trends are consistent for all three predicted outputs. The week 1 parity plots for growth and symmetry, and the corresponding results for tissue context, are included in Supplementary Figs S7 and S8. (B) Line plots show improvement of ML models in both colony and tissue contexts when they are trained on features from timepoints later in the simulation. One-way Dunnet (1955) statistical test with Bonferroni correction show timepoints features that perform better than the initial timepoint. Hash marks signify adjusted *P*-values <.01. (C) Predictive performance as a function of training data for MLP models at later timepoints. These subplots show the average RMSE as a function of the size of training data. The individual points represent the RMSE from randomized test sets.

We found that network metrics that were generated from later timepoints provided better predictive performance (Fig. 3A). In the colony context activity was significantly more predictable when using features representing network properties at later timepoints (Fig. 3B, left). Additionally, we observed nonmonotonic improvements in growth prediction with a notable improvement in performance using snapshots of network properties from the middle of the time course (Fig. 3B, middle, Supplementary Fig. S7). Symmetry showed minimal improvement (Fig. 3B, right). Conversely, in the tissue context, predictions of activity did not improve, while predictions of growth improved monotonically (Fig. 3B, Supplementary Fig. S8). The improvement in predicting symmetry from initial features relative to features from two simulation weeks was larger in tissue contexts than in colony contexts (Fig. 3B, Supplementary Fig. S8).

The analysis of vascular structure included features post-vascular collapse, which likely accounted for some of the better performance. Alternatively, including differential timepoint analyses did not provide additional benefits to predictive power (Supplementary Figs S4 and S5). Simply shortening the simulation time, and therefore the prediction horizon, to one week resulted in a sharp decline in emulator performance (Supplementary Fig. S6). Once again, there was limited improvement in test performance with additional training data, indicating there was sufficient data to train these ML models (Fig. 3C).

### 4.4 Neural network structures trained on network dynamics support ABM emulators

**Figure 4.**
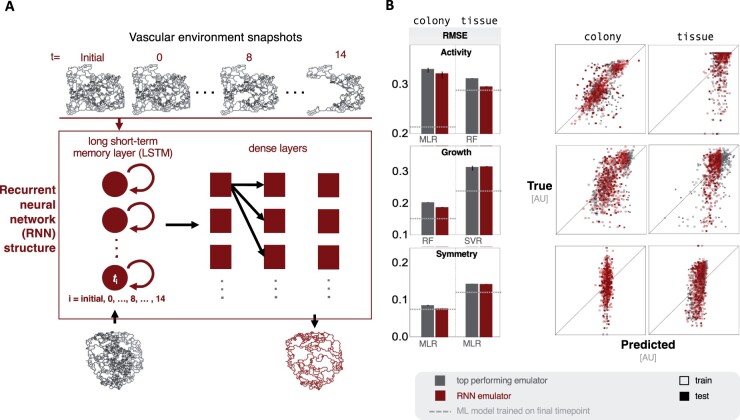
Incorporating temporal information can improve emulation performance—(A) A summary of the vascular network structure and ML model workflow. Network metrics from vascular structures from consecutive simulation days are used as a sequence to train the RNN. Initial vasculatures (left network below RNN structure box) are then used to predict network properties of the vasculatures after two simulation weeks (right network below RNN structure box); the two-week predictions are then used to predict emergent behaviors with ML models. (B) Bar plots show performance between emulators trained on forecasted network metrics and the top performing naive emulation models (noted beneath bars). Associated parity plots show the prediction performance of the RNN models across contexts for each emergent output.

We hypothesized that including temporal dynamics in the training of an emulator could improve performance based on the enhanced predictive potential of later timepoints. First we trained a recurrent NN (RNN) on the network evolution of the vascular architecture in order to forecast the network metrics of the final timepoint based on the initial condition (Fig. 4A). Then, we used statistical learning models to predict emergent outputs using the predicted final network metrics as features (Fig. 4B).

The efficacy of using forecasted features varied widely by emergent outcome and context. Emulators trained on forecasted features never performed worse than those trained on initial-condition-derived features (Fig. 4B, left). In the colony context the RNN model led to a small improvement in activity and growth, while symmetry remained largely unpredictable. In the tissue context, the model was improved across all emergent behaviors. Activity predictions improved in the tissue context with a test $R^{2}$ nearly three times greater than the naive emulation models (Fig. 4B).

While growth exhibited the largest benefits from training on temporal dynamics, all predictions across all emergent outcomes presented at least minor improvements, suggesting that temporal information could benefit the prediction of diverse emergent behaviors. However, these improvement did not match the predictive accuracy of the final timepoints directly from the simulation. Based on a principal component analysis, the features derived from the forecasted network architectures were comparable to those from simulated architectures at the final timepoint, independent of the training-testing split of data (Supplementary Fig. S9). These results indicated that the propagation of minor errors resulting from ML regression techniques may account for the differences in predictive accuracy of the forecasted versus simulated timepoints.

## 5 Discussion

Parity between biological systems and computational models requires algorithms capable of considering higher resolutions of heterogeneous species and their interactions. This objective adds complexity to analysis of experimental data and the development of predictive models. Emulation is a powerful tool that computational scientists can use to reduce the computational expense of model simulations (accelerating hypothesis generation), and to identify and understand key drivers of simulation dynamics (elucidating biological insight). This work unpacks the challenges of parity by using emulation to predict the evolution of tumor development in a dynamic environment that accounts for multilateral regulation among diverse cell agents and between cell agents and their supporting vasculature. The vascular architecture (topology) and function (hemodynamics) change as a function of time and cell population, encompassing a relevant level of biological complexity. While other studies have focused on leveraging emulation to interrogate ABM parameters (Alden *et al.* 2020, Angione *et al.* 2022), we focused on emulators using initial heterogeneous environmental conditions to predict the evolution of the cell population holistically to maintain close analogs with current experimental methodologies (e.g. dynamic imaging).

We built ML models designed to predict emergent behaviors of *in silico* tumors after two simulation weeks. These behaviors include tumor activity, growth, and symmetry. ML model predictions were based on features that derived from a network analysis of the tumor environment’s initial vascular architecture and hemodynamics. Counter-intuitively, we found that spatial characteristics of the environment were largely *un*successful at improving predictions of emergent behaviors that were shown to be associated with spatial phenomena (Yu and Bagheri 2021). The resulting models were prone to both underfitting (evident from poor performance and limited improvement from additional data) and overfitting (indicated by substantial gaps between testing and training splits). Instead, we found that ML models that were trained on environmental snapshots of later simulation timepoints were more predictive of these emergent behaviors.

We then built an emulator using a combination of a RNN-forecaster model to predict the network evolution of the vasculature, and we used these as inputs into ML regression models to predict emergent behaviors. The final RNN-based emulator showed promising, albeit limited, improvement over using emulators that were strictly trained on initial environmental conditions highlighting the role of temporal dynamics in spatio-temporal cell population models. We were able to demonstrate the ability of an RNN model to make accurate forecasts of the network evolution. However, the limited predictive potential of those forecasted timepoints for predicting emergent behavior in turn emphasizes the importance of leveraging ABMs to make more accurate representations of our systems.

By leveraging network analysis and the evolution of network metrics, this study reinforces the importance of temporal information when predicting the behavior of cell population dynamics, even when the emergent behavior derives from spatial dynamics. The difference in performance between our two simulation contexts necessitate careful consideration when translating colony results to tissue contexts—analogous to translating in vitro to in vivo results. A general conclusion of this study suggests that out-of-the-box ML approaches are limited in their ability to characterize spatially heterogeneous systems. Despite their limited performance, we believe that SE would become an invaluable tool to the scientific community once we overcome challenges underlying their predictive performance for general application. Until then, ABMs are a necessary and unmatched alternative to predicting spatio-temporal dynamics.

The emulation of ABMs is difficult due to the complexity (e.g. number of species and corresponding interactions) and emergent nature of the biological phenomena they are well suited to simulate. As ABMs become increasingly multi-scale and complex (Prybutok *et al.* 2022a), challenges in emulation will persist and likely magnify. Mitigating these challenges is necessary to mediate the quantity of ABM simulations required to identify patterns, sample high-dimensional spaces, and generate testable hypotheses across emergent dynamics; such simulations can be cost-prohibitive. This cost is particularly relevant in context of personalized medicine where computational models must be used for real-time control (as is the case of insulin delivery)(Ortmann *et al.* 2019). In order to develop similar interventions that can help mitigate or drive population dynamics, the cost of predicting the spatio-temporal dynamics of cell populations must be addressed head on.

Computational expense remains a significant consideration in supporting emulator development, training, and analysis of perturbations’ impact on outcomes. Furthermore, the high-resolution spatio-temporal data required for emulation necessitates efficient storage, dissemination, and management protocols. The associated computational costs with emulation also call for economic considerations when investigating relationships among state variables or between inputs and outputs, as well as rigorous sampling of an ML model’s hyperparameter space. Additionally, emergent dynamics arising from the evolution of complex spatial and temporal processes poses challenges for representing said data in a ML framework such that the resulting models remain interpretable and useful. These challenges need to be addressed to maximize the potential of SE in advancing our understanding of biological systems through computational modeling.

## Supplementary Material

btae131_Supplementary_Data
