# Supplementary material for: Incorporating temporal information during feature engineering bolsters emulation of spatio-temporal emergence
Source: Bioinformatics. 2024 Mar 5;40(3):btae131. doi: 10.1093/bioinformatics/btae131 (PMC10957516; doi:10.1093/bioinformatics/btae131)
Supplement: btae131_Supplementary_Data [file btae131_supplementary_data.pdf]

Emergent behavior metrics

Emergent behavior metrics are defined and calculated as presented in a previous study of heterogeneous vasculatures in ARCADE (Yu and Bagheri, 2021).

Growth rate

Growth rate quantifies the change in colony diameter over time. First, colony diameter is calculated at each time index. Growth rate is the slope of the simple linear regression between [0, 0.5, ..., t_i] and corresponding diameters [D_0, $D_{0.5}$, ..., D_i], where i indicates the timepoint. These calculations were performed using the Python function `polyfit` from package `numpy` with degree of 1.

Symmetry

Symmetry (S) quantifies colony shape at a given timepoint, ranging from 0 (not symmetric) to 1 (perfectly symmetric). For hexagonal coordinates, a colony is perfectly symmetric if for each location (u,v,w), the corresponding five locations (-w,-u,-v), (v,w,u), (-u,-v,-w), (w,u,v), and (-v,-w,-u) are all occupied. Symmetry is calculated as:

$$S = 1 - \frac{1}{N} \sum_{i}^{N} \frac{n_i}{5}$$

where N is the number of unique locations and n_i is the number of corresponding unoccupied locations for a unique location i.

Activity

Activity (A) quantifies the ratio of active (proliferative and migratory) to inactive (necrotic and apoptotic) cells at a given timepoint. This metric ranges between -1 (all non-quiescent cells are inactive) and +1 (all non-quiescent cells are active). An activity value of 0 indicates an equal number of active and inactive cells. Activity is calculated as:

$$A = 2 * \frac{N_a}{N_a + N_i} - 1$$

where N_a is the number of active cells and N_i is the number of inactive cells.

References

J. S. Yu and N. Bagheri. Modular microenvironment components reproduce vascular dynamics de novo in a multi-scale agent-based model. *Cell Systems*, 12(8):795–809.e9, Aug. 2021. ISSN 2405-4712. doi: 10.1016/j.cels.2021.05.007.

Supp. Table 1. Topological feature list

Feature	Code	Equation	Description
Radius	RADIUS	$\frac{\sum_{e\in E} \text{radius of e}}{\lvert E\rvert}$	Average vessel radius
Length	LENGTH	$\frac{\sum_{e\in E} \text{length of e}}{\lvert E\rvert}$	Average vessel length
Wall	WALL	$\frac{\sum_{e\in E} \text{wall thickness of e}}{\lvert E\rvert}$	Average vessel wall thickness
Shear	SHEAR	$\frac{\sum_{e\in E} \text{shear force on e}}{\lvert E\rvert}$	Average vessel shear force
Circumference	CIRCUM	$\frac{\sum_{e\in E} \text{circumference of e}}{\lvert E\rvert}$	Average vessel circumference
Flow	FLOW	$\frac{\sum_{e\in E} \text{flow through e}}{\lvert E\rvert}$	Average vessel flow
Nodes	NODES	$\lvert V\rvert$	Number of nodes in the graph
Edges	EDGES	$\lvert E\rvert$	Number of edges in the graph
Average eccentricity	AVG_ECCENTRICITY	$\frac{\sum_{v\in V} max(dist(v))}{\lvert V\rvert}$ where $dist(v)$ is the shortest distances between v and all other nodes	The average longest shortest path that each nodes is from another node
Graph radius	GRADIUS	$min(eccentricity)$	The minimum eccentricity value
Graph diameter	GDIAMETER	$max(eccentricity)$	The maximum eccentricity value
Average shortest path	AVG_SHORTEST_PATH	$\frac{\sum_{v\in V} \frac{(dist(v)}{\lvert V\rvert}}{\lvert V\rvert} * \frac{1}{\lvert V\rvert*(\lvert V\rvert-1)}$ where $dist(v)$ is the shortest distances between v and all other nodes	The average shortest path between each nodes and every other node
Average in degree	AVG_IN_DEGREES	$\frac{\sum_{v\in V} indegree(v)}{\lvert V\rvert}$	The average in degree in a directed graph
Average out degree	AVG_OUT_DEGREES	$\frac{\sum_{v\in V} outdegree(v)}{\lvert V\rvert}$	The average out degree in a directed graph
Average degree	AVG_DEGREE	$\frac{\sum_{v\in V} degree(v)}{\lvert V\rvert}$	The average degree in an undirected graph
Average clustering	AVG_CLUSTERING	$\frac{\sum_{v\in V} Cl(v)}{\lvert V\rvert}$ where $Cl(v)$ is the ratio of the ratio between the number of existing connections between neighbors of v and the maximum possible connections between them	The average clustering coefficient of a graph; the tendency for nodes to form tightly interconnected clusters
Average closeness	AVG_CLOSENESS	$\frac{(\lvert V\rvert-1)*(\lvert V\rvert-1)}{\frac{\sum_{v\in V} dist(v)}{\lvert V\rvert}}$ where $dist(v)$ is the shortest distances between v and all other nodes	The average closeness between nodes using the Wasserman and Faust imrpoved formula
Average betweenness	AVG_BETWEENNESS	$\frac{\sum_{v\in V} B(v)}{\lvert V\rvert} * \frac{1}{(\lvert V\rvert-1)*(\lvert V\rvert-2)}$ where $B(v)$ is the sum of the fraction of shortest paths passing through v over all pairs of nodes in the graph	The average betweenness of a graph; the average importance of nodes connectors between other nodes in the network
Average coreness	AVG_CORENESS	$\frac{\sum_{v\in V} C(v)}{\lvert V\rvert}$ where $C(v)$ is the coreness of node v	The average coreness of a graph; the level of connectedness of nodes within a graph

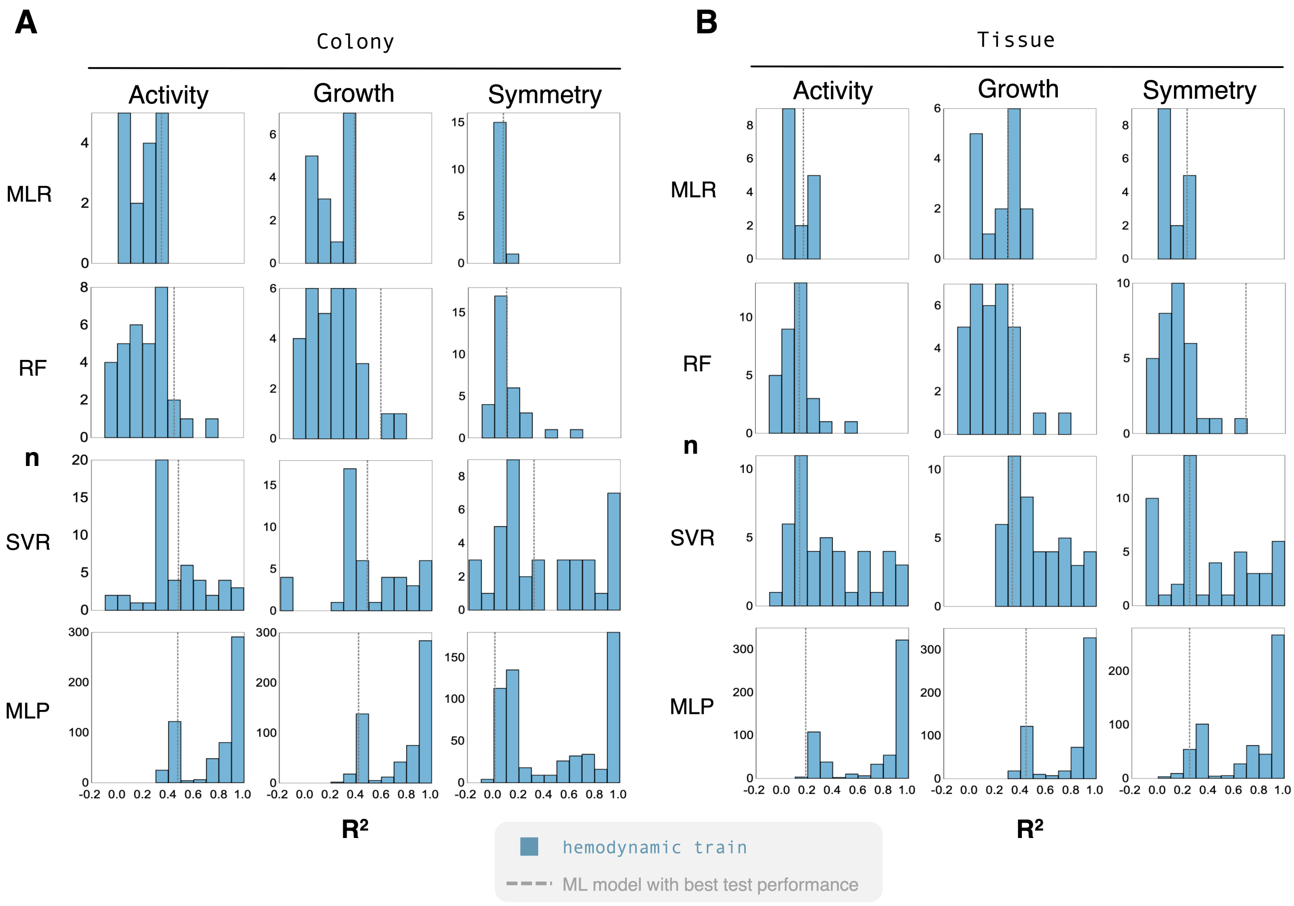

Supp. Fig. 1: **Emulators capture variance across the data during training** — (**A**) Histograms show a range of performances achieved during training for different parameter combinations in the `colony` context. More complex models, such as MLP, can perfectly fit the data in many cases. The vertical dashed line indicates the training performance on model with the best validation performance. (**B**) Similarly, histograms show prediction performance during training across responses and models in the `tissue` context.

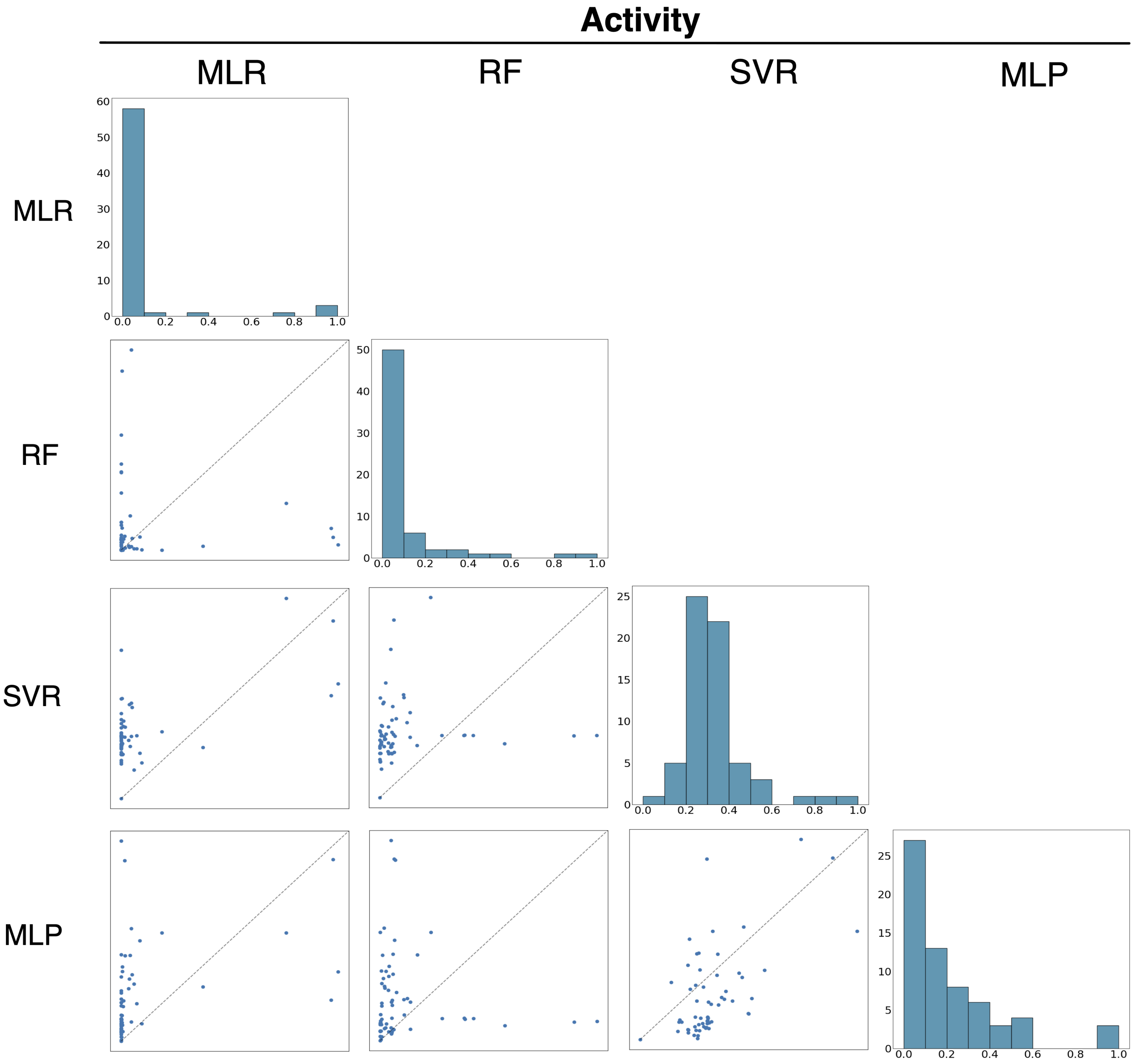

Supp. Fig. 2: **Discrepancy in feature importance across models** — Histograms along the diagonal show the normalized importance distribution of features for each model. Parity plots show the importance of a feature for one model against another. Perfect parity would indicate that the models agreed on the importance level of each feature.

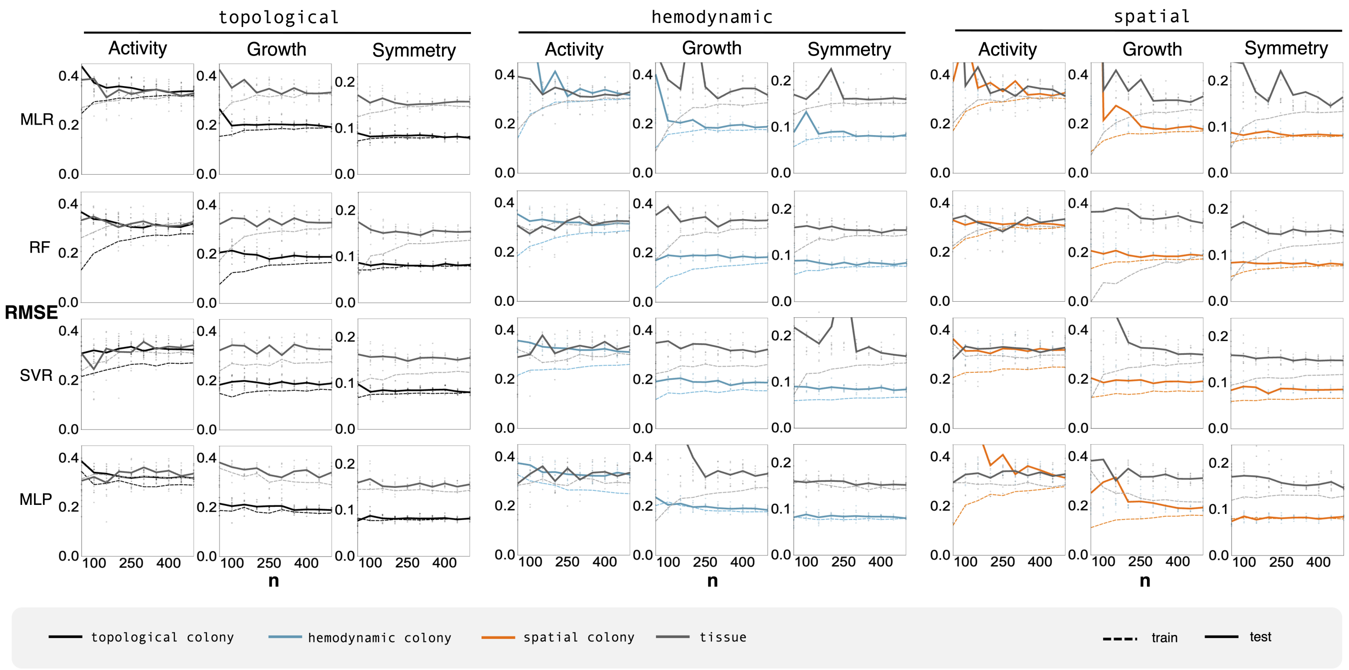

Supp. Fig. 3: **Training data points to diminishing returns** — Line plots indicate the predictive performance of models trained on increasingly large training data sets. In most cases, the RMSE shows diminishing returns of model performance across all feature sets (topolgical, hemodynamic, and spatial), emergent targets (activity, growth, and symmetry), and algorithm (MRL, RF, SVR, MLP).

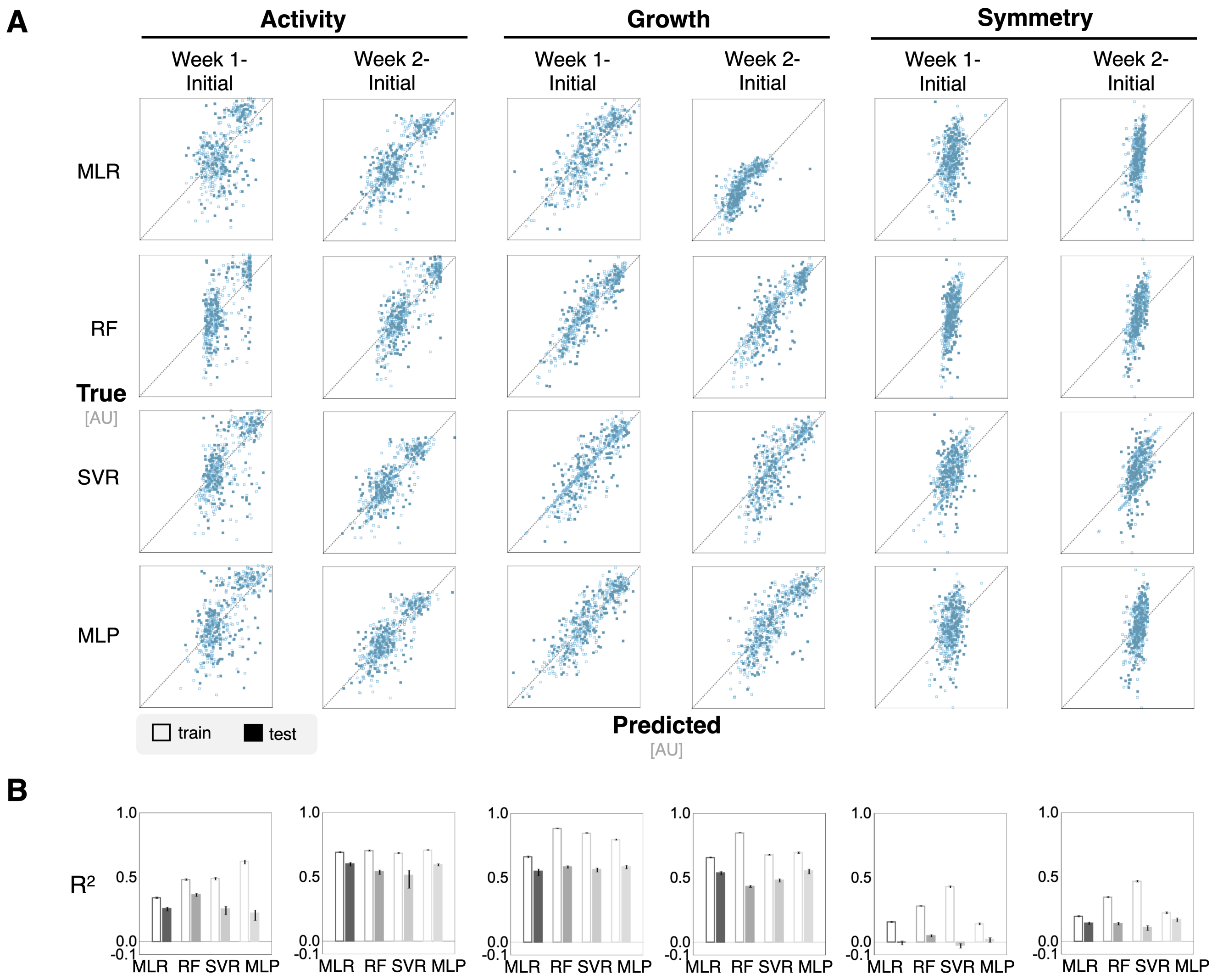

Supp. Fig. 4: **Differential timepoint analysis shows minimal improvement in performance in a `colony` context** — Differential features were calculated by subtracting the features at either one or two weeks from initial features in order to capture the evolution of the features over time. (**A**) Parity plots show the predicted values of emergent targets against the real value to demonstrate fine-grained predictive performance. (**B**) Bar plots show R^2 values for ML models trained on differential features. Growth predictions in the `colony` context improve with simulations using week one and initial timepoint differential features.

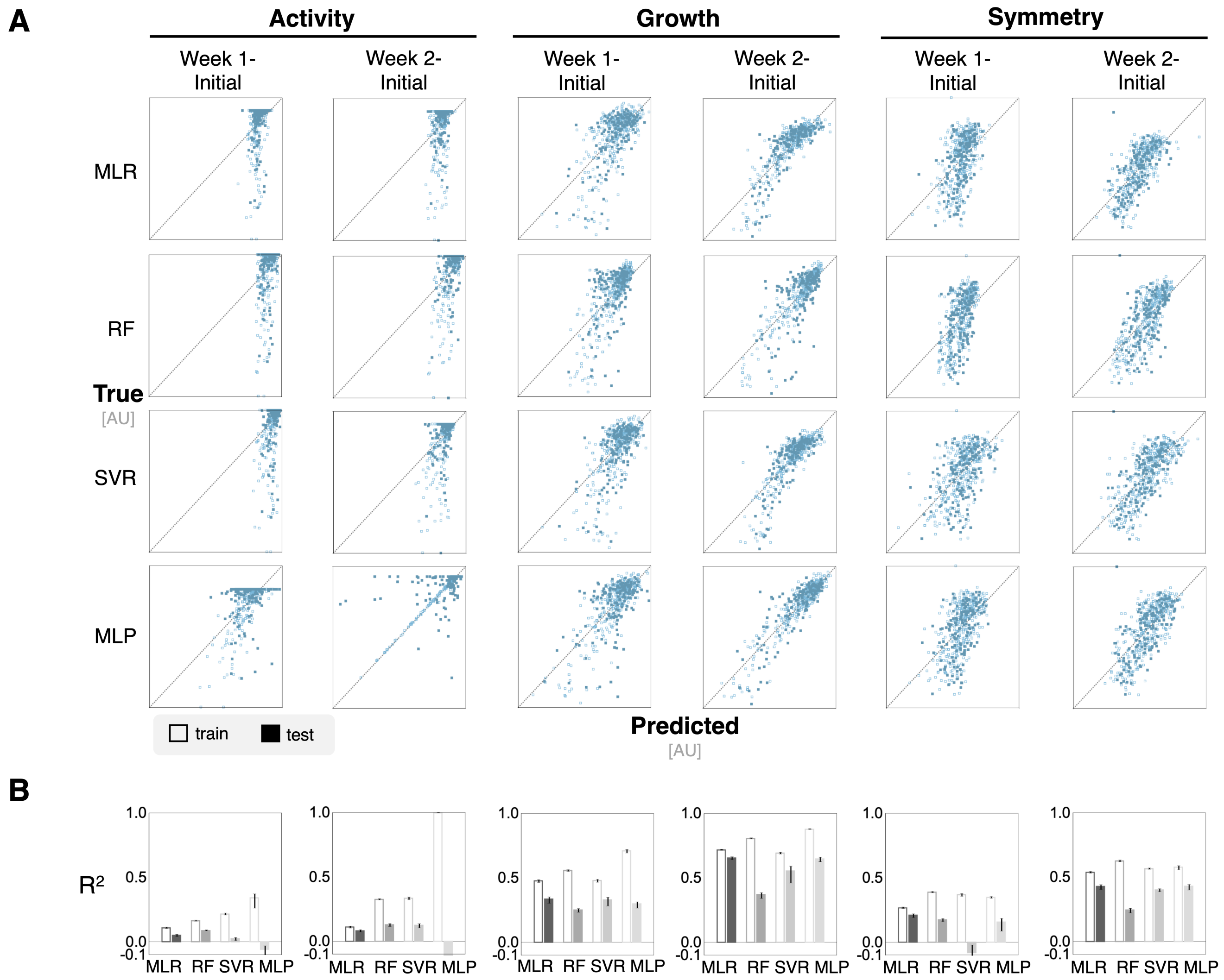

Supp. Fig. 5: **Differential timepoint analysis does not improve performance in a tissue context** — Differential features were calculated by subtracting the features at either one or two weeks from initial features in order to capture the evolution of the features over time. (**A**) Parity plots show the predicted values of emergent targets against the real value to demonstrate fine-grained predictive performance. (**B**) Bar plots show R^2 values for ML models trained on differential features.

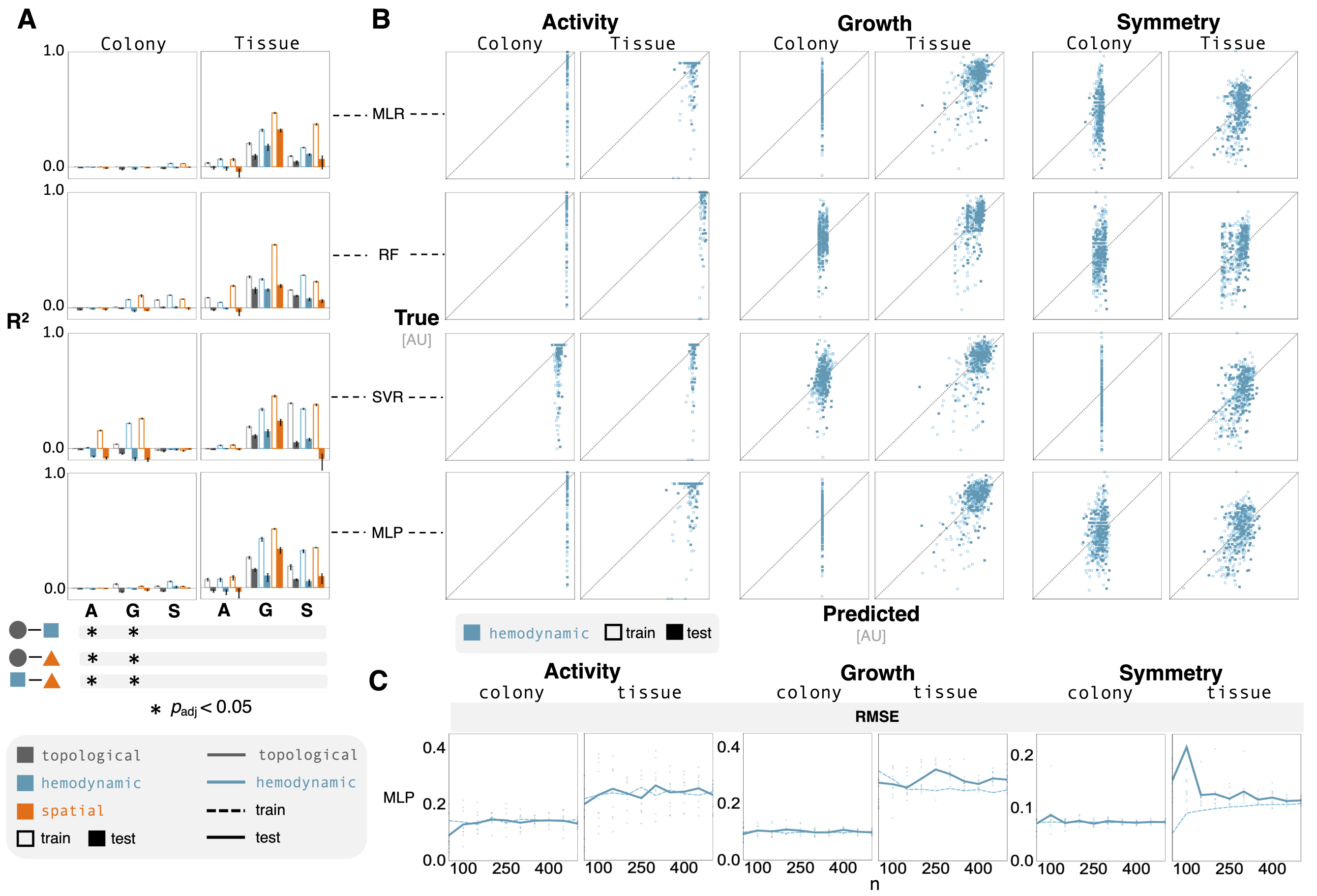

Supp. Fig. 6: **Shortened prediction horizon has minimal effect on emulator performance** — Models predicting spatio-temporal dynamics from after one simulation week. (**A**) Bar plots indicate very poor predictive performance in all cases. Bar chart values range from -0.1 to 1.0; the horizontal axis is at 0.0. The Bonferroni corrected p-values from a two-way ANOVA highlight significant results (noted with black circles) that have an adjusted p-value less that 0.05. (**B**) Parity plots reveal substantial discrepancies in the variance between the predicted and true responses. (**C**) Line plots show predictive performance of the MLP models (the average RMSE) as a function of the size of training data. Performance improvements are limited from additional data. The data points highlight the RMSE from randomized test sets.

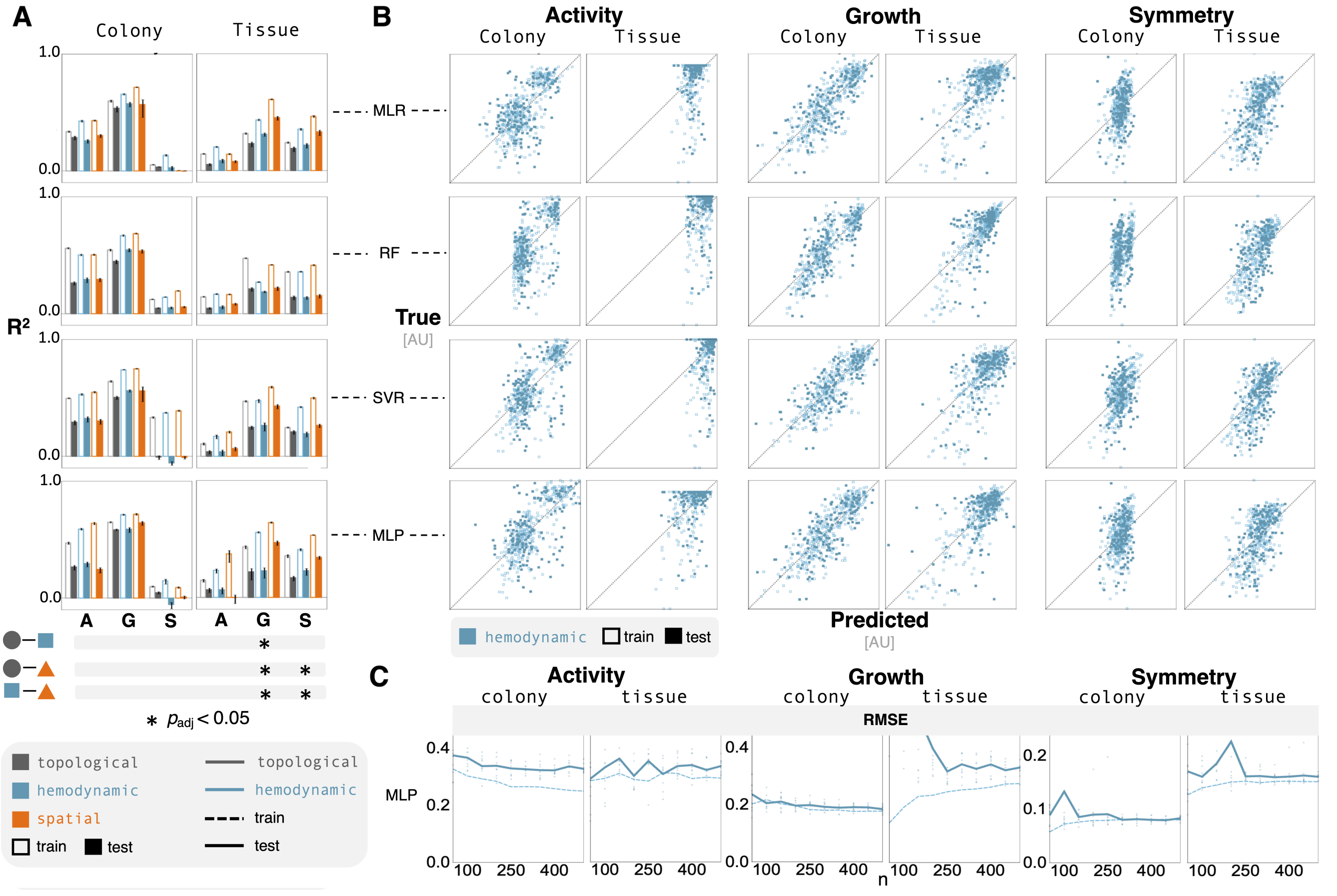

Supp. Fig. 7: **Mid-simulation features show improvement in performance in both contexts** — Models trained on vascular features characterizing network structure at one simulation week result. (**A**) Bar plots show limited improvement from training on mid-simulation features. Bar chart values range from -0.1 to 1.0; the horizontal axis is at 0.0. The Bonferroni corrected p-values from a two-way ANOVA highlight significant results (noted with black circles) that have an adjusted p-value less that 0.05. (**B**) Parity plots reveal large amounts of variance in predicted values with some improvement in growth predictions in the `colony` context. (**C**) Line plots show predictive performance of the MLP models (the average RMSE) as a function of the size of training data. Performance improvements are limited from additional data. The data points highlight the RMSE from randomized test sets.

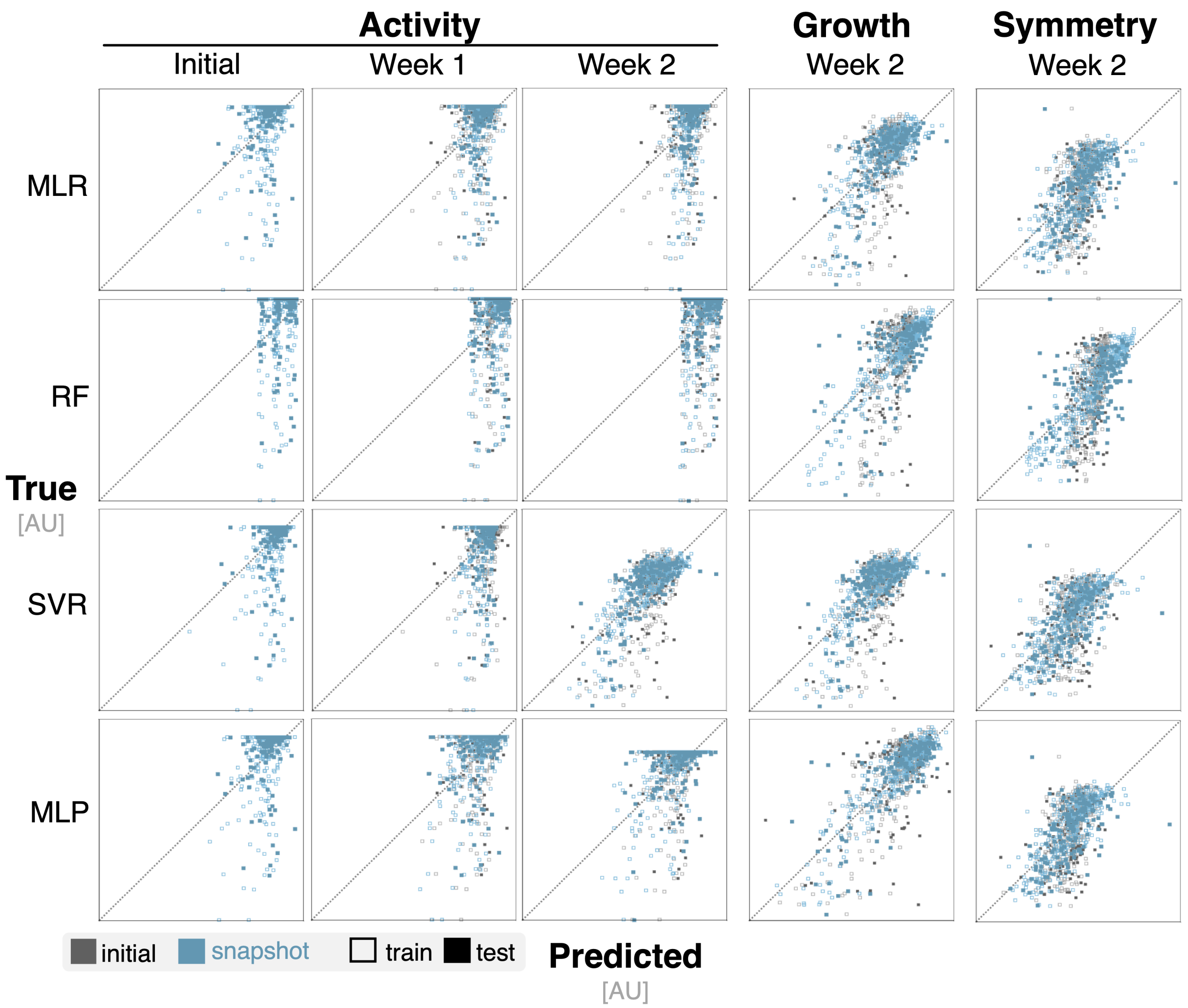

Supp. Fig. 8: **Temporal information improves ML model predictions in `tissue` context** — Parity plots show the predictive performance of ML models trained on features from later timepoints against emulators trained on features from the initial timepoint. Improved prediction of activity is limited; growth and symmetry reflect minimal improvements.

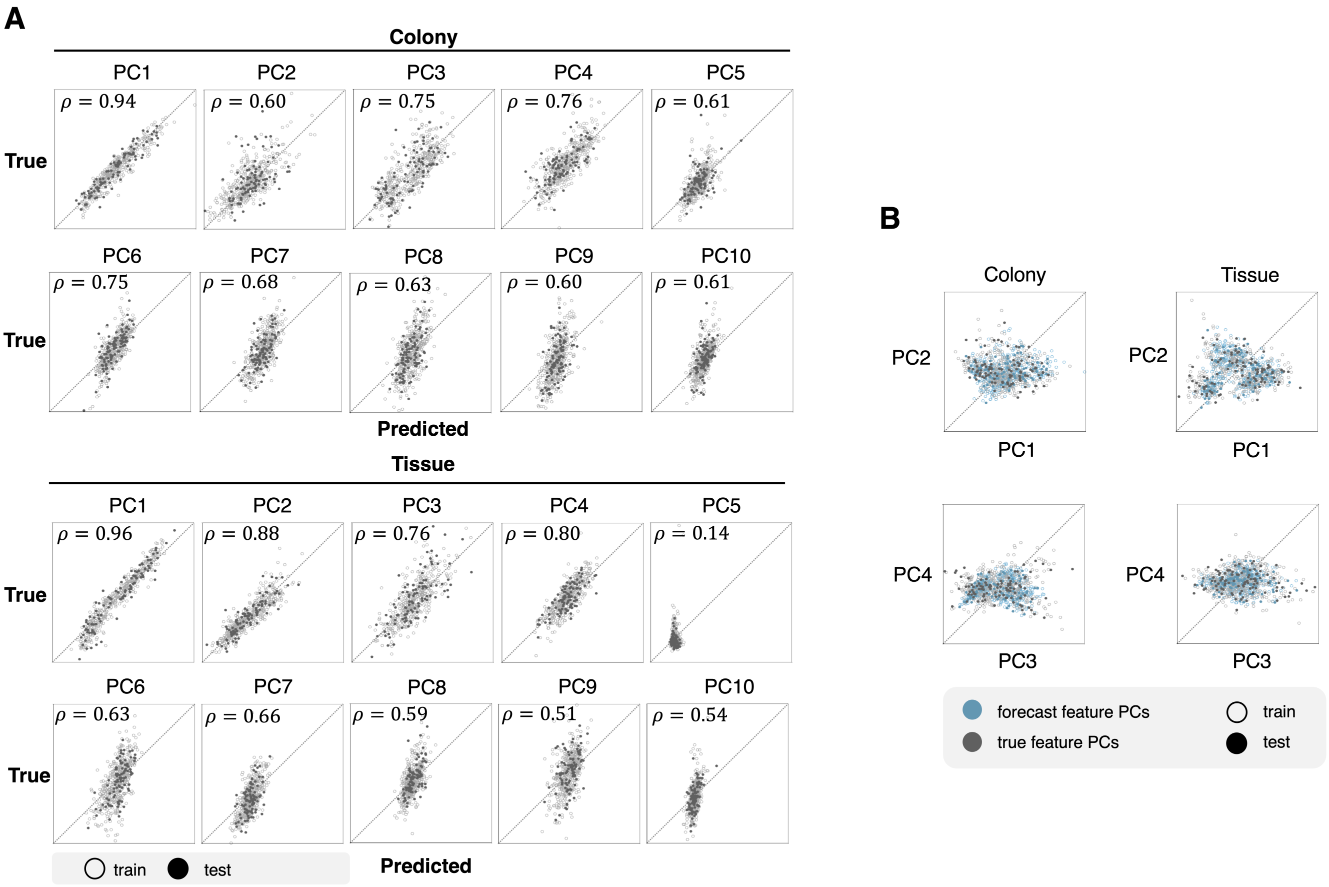

Supp. Fig. 9: **RNN model captures vascular feature variance** — (**A**) Parity plots highlight the performance of a RNN model at predicting network metric features. Both the simulated features and forecasted features were combined to perform PCA. The dimensionality of the feature set are reduced to the first 10 principal components, which represent 95% of the feature variance. The Pearson correlation coefficient (ρ) is reported for each parity plot. (**B**) Scatter plots illustrate the overlap between true and forecasted features using the first four principal components.

Supp. Table 2. Hemodynamic feature list

Feature	Code
Graph radius weighted by flow through the vessel	GRADIUS:FLOW
Graph diameter weighted by flow	GDIAMETER:FLOW
Average eccentricity weighted by flow	AVG_ECCENTRICITY:FLOW
Average shortest path weighted by flow	AVG_SHORTEST_PATH:FLOW
Average closeness weighted by flow	AVG_CLOSENESS:FLOW
Average betweenness weighted by flow	AVG_BETWEENNESS:FLOW
Graph radius weighted by vessel wall thickness	GRADIUS:WALL
Graph diameter weighted by wall	GDIAMETER:WALL
Average eccentricity weighted by wall	AVG_ECCENTRICITY:WALL
Average shortest path weighted by wall	AVG_SHORTEST_PATH:WALL
Average closeness weighted by wall	AVG_CLOSENESS:WALL
Average betweenness weighted by wall	AVG_BETWEENNESS:WALL
Graph radius weighted by vessel shear force	GRADIUS:SHEAR
Graph diameter weighted by shear	GDIAMETER:SHEAR
Average eccentricity weighted by shear	AVG_ECCENTRICITY:SHEAR
Average shortest path weighted by shear	AVG_SHORTEST_PATH:SHEAR
Average closeness weighted by shear	AVG_CLOSENESS:SHEAR
Average betweenness weighted by shear	AVG_BETWEENNESS:SHEAR
Graph radius weighted by vessel radius	GRADIUS:RADIUS
Graph diameter weighted by radius	GDIAMETER:RADIUS
Average eccentricity weighted by radius	AVG_ECCENTRICITY:RADIUS
Average shortest path weighted by radius	AVG_SHORTEST_PATH:RADIUS
Average closeness weighted by radius	AVG_CLOSENESS:RADIUS
Average betweenness weighted by radius	AVG_BETWEENNESS:RADIUS
Graph radius weighted by average pressure across each vessel	GRADIUS:PRESSURE_AVG
Graph diameter weighted by average pressure	GDIAMETER:PRESSURE_AVG
Average eccentricity weighted by average pressure	AVG_ECCENTRICITY:PRESSURE_AVG
Average shortest path weighted by average pressure	AVG_SHORTEST_PATH:PRESSURE_AVG
Average closeness weighted by average pressure	AVG_CLOSENESS:PRESSURE_AVG
Average betweenness weighted by average pressure	AVG_BETWEENNESS:PRESSURE_AVG
Graph radius weighted by pressure delta across each vessel	GRADIUS:PRESSURE_DELTA
Graph diameter weighted by pressure delta	GDIAMETER:PRESSURE_DELTA
Average eccentricity weighted by pressure delta	AVG_ECCENTRICITY:PRESSURE_DELTA
Average shortest path weighted by pressure delta	AVG_SHORTEST_PATH:PRESSURE_DELTA
Average closeness weighted by pressure delta	AVG_CLOSENESS:PRESSURE_DELTA
Average betweenness weighted by pressure delta	AVG_BETWEENNESS:PRESSURE_DELTA
Graph radius weighted by average oxygen across each vessel	GRADIUS:OXYGEN_AVG
Graph diameter weighted by average oxygen	GDIAMETER:OXYGEN_AVG
Average eccentricity weighted by average oxygen	AVG_ECCENTRICITY:OXYGEN_AVG
Average shortest path weighted by average oxygen	AVG_SHORTEST_PATH:OXYGEN_AVG
Average closeness weighted by average oxygen	AVG_CLOSENESS:OXYGEN_AVG
Average betweenness weighted by average oxygen	AVG_BETWEENNESS:OXYGEN_AVG
Graph radius weighted by oxygen delta across each vessel	GRADIUS:OXYGEN_DELTA
Graph diameter weighted by oxygen delta	GDIAMETER:OXYGEN_DELTA
Average eccentricity weighted by oxygen delta	AVG_ECCENTRICITY:OXYGEN_DELTA
Average shortest path weighted by oxygen delta	AVG_SHORTEST_PATH:OXYGEN_DELTA
Average closeness weighted by oxygen delta	AVG_CLOSENESS:OXYGEN_DELTA
Average betweenness weighted by oxygen delta	AVG_BETWEENNESS:OXYGEN_DELTA

Supp. Table 3. Spatial feature list

Feature	Code
Graph radius weighted by inverse distance	`GRADIUS:INVERSE_DISTANCE`
Graph diameter weighted by inverse distance	`GDIAMETER:INVERSE_DISTANCE`
Average eccentricity weighted by inverse distance	`AVG_ECCENTRICITY:INVERSE_DISTANCE`
Average shortest path weighted by inverse distance	`AVG_SHORTEST_PATH:INVERSE_DISTANCE`
Average closeness weighted by inverse distance	`AVG_CLOSENESS:INVERSE_DISTANCE`
Average betweenness weighted by inverse distance	`AVG_BETWEENNESS:INVERSE_DISTANCE`
Average eccentricity weighted by distance	`AVG_ECCENTRICITY_WEIGHTED`
Average closeness weighted by distance	`AVG_CLOSENESS_WEIGHTED`
Average coreness weighted by distance	`AVG_CORENESS_WEIGHTED`
Average betweenness weighted by distance	`AVG_BETWEENNESS_WEIGHTED`
Average in degree weighted by distance	`AVG_IN_DEGREES_WEIGHTED`
Average out degree weighted by distance	`AVG_OUT_DEGREES_WEIGHTED`
Average degree weighted by distance	`AVG_DEGREE_WEIGHTED`

Supp. Table 4. Emulator run times across feature sets on an m5.large EC2 instance

Model	Feature set	Time (m) $\pm$ S.D.
MLR	Topological	0.21 $\pm$ 0.01
	Hemodynamic	1.45 $\pm$ 0.03
	Spatial	1.63 $\pm$ 0.04
RF	Topological	1.11 $\pm$ 0.01
	Hemodynamic	2.51 $\pm$ 0.26
	Spatial	2.78 $\pm$ 0.08
SVR	Topological	1.07 $\pm$ 0.01
	Hemodynamic	3.62 $\pm$ 0.06
	Spatial	3.68 $\pm$ 0.05
MLP	Topological	128.62 $\pm$ 1.61
	Hemodynamic	285.71 $\pm$ 1.18
	Spatial	286.20 $\pm$ 0.77

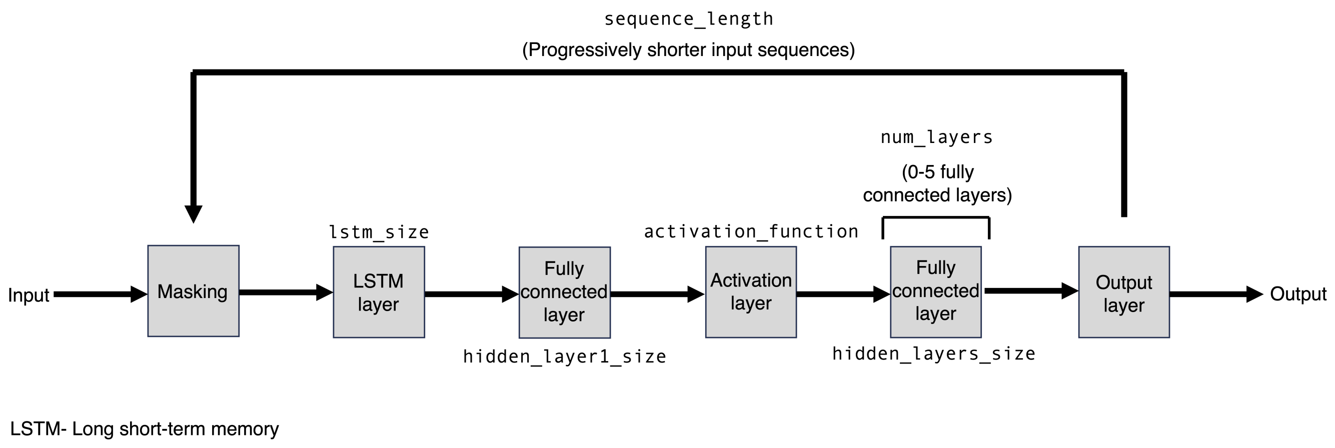

Supp. Fig. 10: **Architecture and structure of the RNN used for feature prediction** — This flowchart describes the layers used while training the feature prediction RNN. All parameter values are defined in Supp. Table 7 and optimal values are provided in Supp. Table 8.

Supp. Table 5. Top performing hyperparameters for emulation models in a colony context

Model	Emergent Target	Feature set	Hyperparameters
MLR	Activity	Topological	alpha:0.001;l1_ratio:0.1;max_iter:10000
		Hemodynamic	alpha:0.0023; l1_ratio:0.6625; max_iter:10000
		Spatial	alpha:0.0015; l1_ratio:0.9437; max_iter:10000
	Growth	Topological	alpha:0.001; l1_ratio:0.1 ; max_iter:10000
		Hemodynamic	alpha:0.0036; l1_ratio:0.3812; max_iter:10000
		Spatial	alpha:0.0036; l1_ratio:0.3812; max_iter:10000
	Symmetry	Topological	alpha:0.0056; l1_ratio:0.775; max_iter:10000
		Hemodynamic	alpha:0.0036; l1_ratio:0.3812; max_iter:10000
		Spatial	alpha:0.0316; l1_ratio:0.55; max_iter:10000
RF	Activity	Topological	n_estimators:13; max_features:0.1778; max_depth:38; min_samples_split:0.0177; min_samples_leaf:0.0177; bootstrap:True
		Hemodynamic	n_estimators:81; max_features:0.2371; max_depth:81; min_samples_split:0.0133; min_samples_leaf:0.0749; bootstrap:False
		Spatial	n_estimators:81; max_features:0.2371; max_depth:81; min_samples_split:0.0133; min_samples_leaf:0.0749; bootstrap:False
	Growth	Topological	n_estimators:26; max_features:0.3162; max_depth:75; min_samples_split:0.3162; min_samples_leaf:0.0316; bootstrap:False
		Hemodynamic	n_estimators:81; max_features:0.2371; max_depth:81; min_samples_split:0.0133; min_samples_leaf:0.0749; bootstrap:False
		Spatial	n_estimators:81; max_features:0.2371; max_depth:81; min_samples_split:0.0133; min_samples_leaf:0.0749; bootstrap:False
	Symmetry	Topological	n_estimators:57; max_features:0.0749; max_depth:7; min_samples_split:0.4216; min_samples_leaf:0.0237; bootstrap:False
		Hemodynamic	n_estimators:20; max_features:0.0421; max_depth:94; min_samples_split:0.0749; min_samples_leaf:0.1333; bootstrap:False
		Spatial	n_estimators:94; max_features:0.0133; max_depth:69; min_samples_split:0.0237; min_samples_leaf:0.04216; bootstrap:True
SVR	Activity	Topological	C:0.5623; epsilon:0.1778; kernel:rbf
		Hemodynamic	C:0.5623; epsilon:0.1778; kernel:rbf
		Spatial	C:0.5623; epsilon:0.1778; kernel:rbf
	Growth	Topological	C:0.1; epsilon:0.001; kernel:rbf
		Hemodynamic	C:0.2371; epsilon:0.0749; kernel:rbf
		Spatial	C:0.2371; epsilon:0.0749; kernel:linear
	Symmetry	Topological	C:0.2371; epsilon:0.0749; kernel:rbf
		Hemodynamic	C:0.2371; epsilon:0.07498; kernel:rbf
		Spatial	C:0.2371; epsilon:0.0749; kernel:rbf
MLP	Activity	Topological	alpha:0.3162; activation:logistic; hidden_layer_sizes:[50, 25]; solver:lbfgs; max_iter:1000
		Hemodynamic	alpha:0.5623; activation:logistic; hidden_layer_sizes:[25, 50]; solver:lbfgs; max_iter:1000
		Spatial	alpha:0.5623; activation:logistic; hidden_layer_sizes:[25, 50]; solver:lbfgs; max_iter:1000
	Growth	Topological	alpha:0.1778; activation:logistic; hidden_layer_sizes:[25, 25]; solver:lbfgs; max_iter:1000
		Hemodynamic	alpha:0.5623; activation:logistic; hidden_layer_sizes:[5]; solver:lbfgs; max_iter:1000
		Spatial	alpha:0.3162; activation:logistic; hidden_layer_sizes:[25, 25]; solver:lbfgs; max_iter:1000
	Symmetry	Topological	alpha:0.3162; activation:logistic; hidden_layer_sizes:[50]; solver:lbfgs; max_iter:1000
		Hemodynamic	alpha:0.0005; activation:identity; hidden_layer_sizes:[5, 10]; solver:lbfgs; max_iter:1000
		Spatial	alpha:0.1778; activation:logistic; hidden_layer_sizes:[50, 50]; solver:lbfgs; max_iter:1000

Supp. Table 6. Top performing hyperparameters for emulation models in a `tissue` context

Model	Emergent Target	Feature set	Hyperparameters
`MLR`	Activity	Topological	alpha:0.0086; l1_ratio:0.2687; max_iter:10000
		Hemodynamic	alpha:0.0056; l1_ratio:0.775; max_iter:10000
		Spatial	alpha:0.0749; l1_ratio:0.2125; max_iter:10000
	Growth	Topological	alpha:0.0056; l1_ratio:0.775; max_iter:10000
		Hemodynamic	alpha:0.0133; l1_ratio:0.4375; max_iter:10000
		Spatial	alpha:0.0023; l1_ratio:0.6625; max_iter:10000
	Symmetry	Topological	alpha:0.0015; l1_ratio:0.9437; max_iter:10000
		Hemodynamic	alpha:0.0015; l1_ratio:0.9437; max_iter:10000
		Spatial	alpha:0.0015; l1_ratio:0.9437; max_iter:10000
`RF`	Activity	Topological	n_estimators:38; max_features:0.0562; max_depth:63; min_samples_split:0.5623; min_samples_leaf:0.0562; bootstrap:False
		Hemodynamic	n_estimators:38; max_features:0.0562; max_depth:63; min_samples_split:0.5623; min_samples_leaf:0.0562; bootstrap:False
		Spatial	n_estimators:20; max_features:0.0421; max_depth:94; min_samples_split:0.0749; min_samples_leaf:0.1333; bootstrap:False
	Growth	Topological	n_estimators:13; max_features:0.1778; max_depth:38; min_samples_split:0.0177; min_samples_leaf:0.0177; bootstrap:True
		Hemodynamic	n_estimators:57; max_features:0.0749; max_depth:7; min_samples_split:0.4216; min_samples_leaf:0.0237; bootstrap:True
		Spatial	n_estimators:81; max_features:0.2371; max_depth:81; min_samples_split:0.0133; min_samples_leaf:0.0749; bootstrap:False
	Symmetry	Topological	n_estimators:94; max_features:0.0133; max_depth:69; min_samples_split:0.0237; min_samples_leaf:0.0421; bootstrap:False
		Hemodynamic	n_estimators:94; max_features:0.0133; max_depth:69; min_samples_split:0.0237; min_samples_leaf:0.0421; bootstrap:False
		Spatial	n_estimators:81; max_features:0.2371; max_depth:81; min_samples_split:0.0133; min_samples_leaf:0.0749; bootstrap:False
`SVR`	Activity	Topological	C:0.5623; epsilon:0.1778; kernel:linear
		Hemodynamic	C:0.2371; epsilon:0.0749; kernel:linear
		Spatial	C:0.2371; epsilon:0.0749; kernel:rbf
	Growth	Topological	C:0.5623; epsilon:0.1778; kernel:rbf
		Hemodynamic	C:1.333; epsilon:0.0133; kernel:linear
		Spatial	C:4.8696; epsilon:0.0205; kernel:linear
	Symmetry	Topological	C:0.2371; epsilon:0.0749; kernel:linear
		Hemodynamic	C:0.2371; epsilon:0.0749; kernel:linear
		Spatial	C:0.1; epsilon:0.001; kernel:linear
`MLP`	Activity	Topological	alpha:0.3162; activation:logistic; hidden_layer_sizes:[50, 25]; solver:lbfgs; max_iter:1000
		Hemodynamic	alpha:0.5623; activation:logistic; hidden_layer_sizes:[50, 25]; solver:lbfgs; max_iter:1000
		Spatial	alpha:0.5623; activation:logistic; hidden_layer_sizes:[50, 25]; solver:lbfgs; max_iter:1000
	Growth	Topological	alpha:0.5623; activation:logistic; hidden_layer_sizes:[50]; solver:lbfgs; max_iter:1000
		Hemodynamic	alpha:0.5623; activation:identity; hidden_layer_sizes:[25]; solver:lbfgs; max_iter:1000
		Spatial	alpha:0.5623; activation:identity; hidden_layer_sizes:[5]; solver:lbfgs; max_iter:1000
	Symmetry	Topological	alpha:0.5623; activation:tanh; hidden_layer_sizes:[50, 50]; solver:lbfgs; max_iter:1000
		Hemodynamic	alpha:0.5623; activation:identity; hidden_layer_sizes:[5, 5]; solver:lbfgs; max_iter:1000
		Spatial	alpha:0.5623; activation:identity; hidden_layer_sizes:[5, 10]; solver:lbfgs; max_iter:1000

Supp. Table 7. Hyperparameters used in RNN architecture grid search

Hyperparameter	Code	Potential value
Training sequence lengths	sequence_length	[15, 10, 5, 3, 1]]
		[15, 10, 5, 1]
		[15, 5, 3, 1]
LSTM layer size	lstm_size	64
		128
		256
		512
		1024
Size of first hidden layer	hidden_layer1_size	64
		128
		256
		512
		1024
Size of the remaining hidden layers	hidden_layers_size	64
		128
		256
		512
		1024
Number of hidden layers after the first	num_layers	0
		1
		2
		3
		4
		5
Activation function after first hidden layer	activation_function	`relu`
		`tanh`

Supp. Table 8. Top performing architecture parameters for RNN

Context	Parameter	Value
colony	`sequence_length`	[15, 10, 5, 3, 1]
	`lstm_size`	512
	`hidden_layer1_size`	1024
	`hidden_layers_size`	1024
	`num_layers`	2
	`activation_function`	`relu`
tissue	`sequence_length`	[15, 10, 5, 3, 1]
	`lstm_size`	512
	`hidden_layer1_size`	512
	`hidden_layers_size`	1024
	`num_layers`	1
	`activation_function`	`relu`
